# Field evaluation of a schistosome circulating cathodic antigen rapid test kit at point-of-care for mapping of schistosomiasis endemic districts in The Gambia

**DOI:** 10.1371/journal.pone.0182003

**Published:** 2017-08-10

**Authors:** Bakary Sanneh, Ebrima Joof, Abdoulie M. Sanyang, Kristen Renneker, Yaya Camara, Alhagie Papa Sey, Sheriffo Jagne, Ignatius Baldeh, Serign Jawo Ceesay, Sana M. Sambou, Kisito Ogoussan

**Affiliations:** 1 National Public Health Laboratories, Ministry of Health and Social Welfare, Banjul, The Gambia; 2 Neglected Tropical Diseases Support Center, Decatur, Georgia, United States of America; 3 Epidemiology and disease Control Department, Ministry of Health and Social Welfare, Banjul, The Gambia; 4 Medical Research Council, Atlantic Boulevard, Fajara, The Gambia; Food and Drug Administration, UNITED STATES

## Abstract

**Background:**

Studies in Sub Saharan Africa have shown that the Circulating Cathodic Antigen point-of-care-test (POC-CCA) is more accurate in the detections of *S*. *mansoni* than the microscopic Kato-Katz technique but less is known about the accuracy of this rapid test in detecting *S*. *haematobium* infections. This study was intended to evaluate the field accuracy of POC-CCA as a rapid test kit for schistosomiasis mapping in The Gambia.

**Methods:**

This prospective study was conducted in 4 regions in the country. Ten schools were randomly selected from each region, and a total of 2018 participants whose ages range from 7 to 14 years were enrolled in the study. Stool and urine samples were collected from each participant from May to June 2015, and tested for *S*. *haematobium* and *S*. *mansoni* infections in field and laboratory settings. The tests conducted included POC-CCA, double Kato-Katz slides, urine filtration and dipstick for hematuria.

**Results:**

Of the 1954 participants that had complete data, the mean age of participants was 9.9 years. The prevalence of children infected with *S*. *haematobium*, using urine filtration technique was 10.1% (95% CI: 8.87–11.55). Central River Region had the highest level of urinary schistosomiasis with a prevalence of 28.0% (24.13–32.12).The lowest urinary schistosomiasis prevalence of 0.6% (0.12–1.86) was found in Lower River Region and North Bank Region had no cases of schistosomiasis detected. Only 5 participants were infected with *S*. *mansoni*. Using urine filtration as reference standard for the detection of *S*. *haematobium*, the sensitivity and specificity of POC-CCA was 47.7% and 75.8%. Whilst sensitivity and specificity of POC-CCA for detecting *S*. *mansoni* were 60.0% and 71.2% using double Kato-Katz as reference standard.

**Conclusion:**

This study showed lower sensitivity of POC-CCA in detecting *S*. *haematobium*. Therefore POC-CCA is less useful for rapid diagnosis of urinary schistosomiasis.

## Background

Schistosomiasis is a chronic infection endemic in over 74 tropical and sub-tropical countries; more than 200 million people are infected and 650 million people thought to be at risk[1]. Sub-Saharan Africa carries the highest burden (90%) of schistosomiasis and both *S*. *mansoni* and *S*. *haematobium* infections are prevalent [1]. Schistosomiasis is caused by trematode parasites belonging to the genus Schistosoma with S. haematobium, S. mansoni, and S. japonicum representing the species that typically infect humans. The move to control schistosomiasis and other NTDs has gained momentum after the London declaration of 30^th^ January 2012, which advocated for the elimination of schistosomiasis in some countries [2, 3]. World Health Assembly Resolution WHA 65.21 has resulted in scaled up of efforts worldwide to control morbidity of the disease so that the target for elimination can be met. Preventive chemotherapy, or mass drug administration (MDA), using praziquantel is the recommended method of control for schistosomiasis by the World Health Organization (WHO). Before the implementation of MDA campaigns, infection prevalence should be assessed to guide program decision making. This is usually achieved through surveys based on the use of traditional parasitological methods (urine filtration for *S*. *haematobium* and Kato-Katz thick smears for *S*. *mansoni* infections) [4]. These methods are useful for detecting active infection, but are insensitive and often miss light-intensity infections [5, 6]. This resulted to increased efforts to develop more sensitive methods, including molecular techniques and more rapid techniques such as the point- of- care (POC) test for circulating cathodic antigen (CCA) [7, 8, 9] Traditional parasitological methods of diagnosis (urine filtration and Kato-Katz) are useful in areas of high endemicity. However, they are less sensitive for diagnosis in populations where schistosomiasis prevalence is low [6, 10]. Since preventive chemotherapy-based schistosomiasis control programmes are due to be scaled up [11], low infection intensity is expected in areas where MDA campaigns are successful. More sensitive methods of diagnosis will be needed to monitor prevalence and intensity of infection following MDA. Furthermore, the WHO has recently recommended that preschool-aged children be included in treatment programmes [12]. A significant proportion of these children harbor light infections [13] and are therefore more likely to be missed by traditional parasitological methods.

POC-CCA is a rapid, sensitive, user-friendly, equipment-free and deliverable tool and it has been found that a single POC-CCA was nearly three times more sensitive than four Kato-Katz preparations for the diagnosis of *S*. *mansoni* [9]. However, studies have found that POC-CCA was less sensitive for the diagnosis of *S*. *haematobium* [9,14,15]. However, more evaluation studies on the field sensitivity of POC-CCA as a rapid mapping tool for schistosomiasis are recommended [15]. In The Gambia, there is limited information on the current endemicity of schistosomiasis [16,17, 18,19]. Neither historical nor clinical data provide reliable and adequate information on the endemicity of schistosomiasis in The Gambia, thus, new surveys are needed[19,20].This study evaluated the field accuracy of POC-CCA as tool for mapping of schistosomiasis endemic regions in the country.

## Materials and methods

### Ethical issues

The study was approved by the Gambia Government and Medical Research Council Joint Ethical Committee with a number of SCC1415. Written consent forms were signed on behalf of all the students whose parents or guardians were contacted by the head master/mistress. In addition, informed consent was obtained from each study participant as they were enrolled by the data collection teams.

### Study design

The survey was a prospective and cross-sectional study that assessed the sensitivity and specificity of the POC-CCA compared to traditional tools in determining the prevalence of schistosomiasis.

### Study site, population and participant selection

**Fig 1:**
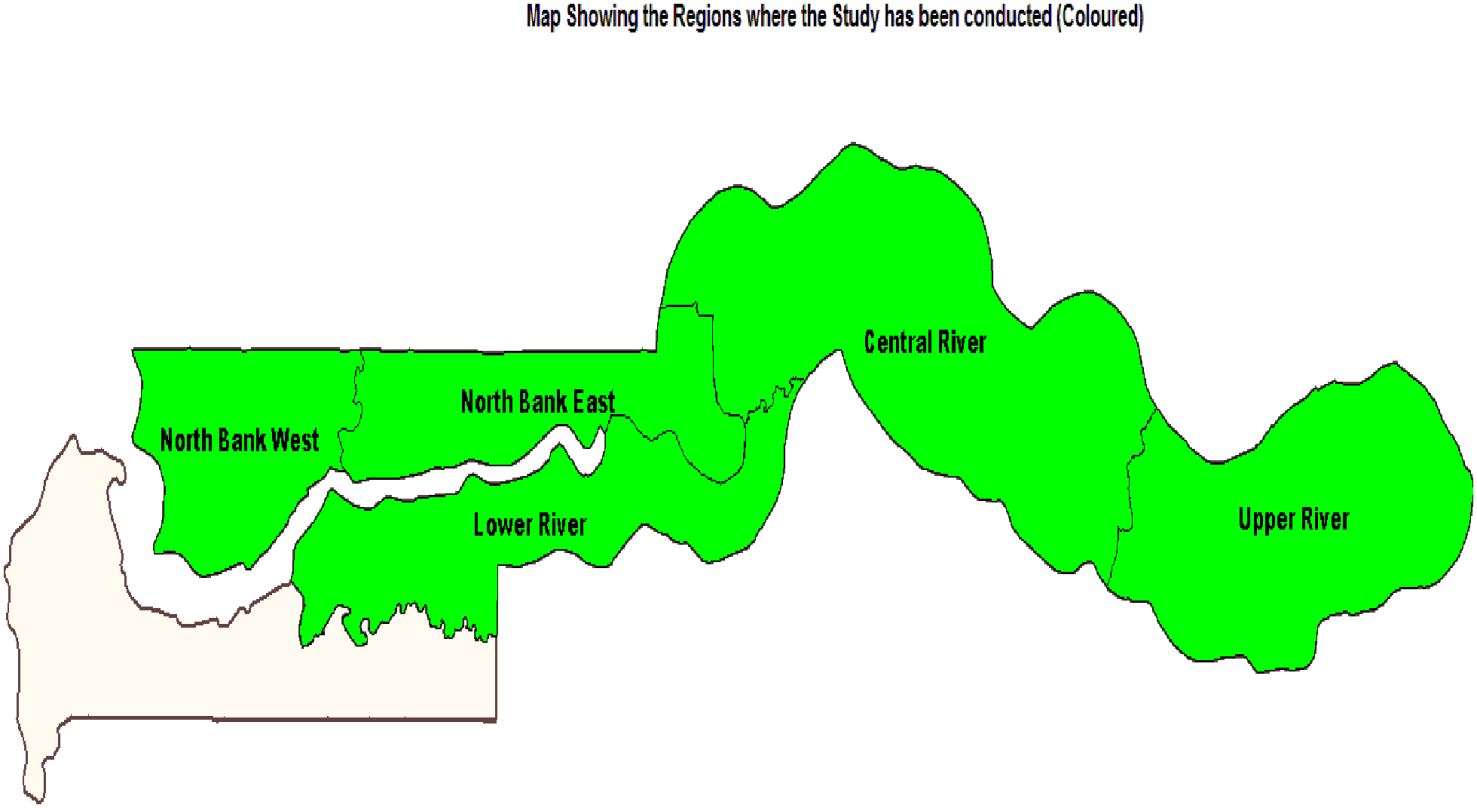
A map showing the four regions (coloured) in which the study had been conducted.

The study was conducted in 4 regions across the country, namely: North Bank Region (NBR), Lower River Region (LRR), Central River Region (CRR) and Upper River Region (URR) (Fig 1). Ten schools were randomly selected from each region, including CRR and URR, which are the historically high endemic regions of schistosomiasis, LRR which has moderate endemicity and NBR which was considered to be non-endemic. Fifty students (25 boys and 25 girls) between the age of 7 and 14 years were randomly selected from each school. In total, 2018 children from 40 schools were enrolled for the study. Stool, urine and blood samples were collected from each participant; 1954 participants have complete data record and included for the analysis. The sample size determination was based on the WHO recommended protocols with modifications for NTD mapping in the WHO AFRO Region Enrolled participants were students who lived in the selected district and attended the selected school, were between 7and 14 years of age, provide informed consent and submitted all requested specimens. Students absent from school during the study period were excluded from the study.

### Sample collection

To obtain consent, head teachers in schools were informed and obtained consent from parents, guardians or wards of the students prior to the survey exercise. Written consent forms were signed on behalf of all the students whose parents or guardians were contacted by the head master/mistress. In addition, informed consent was obtained from each study participant as they were enrolled by the data collection teams. Participants who gave their consent were given two containers to voluntarily donate their urine and stool samples, which were collected in the morning between the hours 8:00am and 12:00 mid-day.

### Parasitological investigations

Each participant’s urine sample was analyzed on site using POC-CCA according to manufacturer’s instructions[21]. Two Kato-Katz slides were prepared from each stool sample and a examine with a microscopic to *S*. *mansoni* eggs. Also, each urine samples was adequately shaken before 10 ml being passed through the urine filtration kit and the filter transferred to a slide for microscopic examination for *S*. *heamatobium* eggs. Haematuria level was investigated using a rapid urine dipstick (Hemastix) for the samples.

### Data analysis

Prevalence estimates with 95% CIs and 2x2 tables comparing POC-CCA results to Kato-Katz, urine filtration, and dipstick methods were computed using SAS University Edition. Due to the proportions being sometimes close to 0, the Agresti-Coull method was used to calculate 95% CIs.

## Results

### Population characteristics and prevalence of schistosomiasis

A total of 2018 participants were enrolled in the study. 1954 participants had complete data that was included in the final analysis (Table 1). The final sample included 975 boys and 979 girls between the ages of 7 to 14 years from across the four regions of the study area. The mean age of participants was 9.9 ± 0.05 years.

**Table 1:** Population characteristics and prevalence of schistosomiasis.

		Filtration		KK		POC-CCA		Dipstick	
Region	no. children investigated	no. infected	prevalence of infection % (95% CI)	no. infected	prevalence of infection % (95% CI)	no. infected	prevalence of infection % (95% CI)	no. infected	prevalence of infection % (95% CI)
CRR	483	135	27.95 (24.13–32.12)	4	0.83 (0.24–2.19)	147	30.43 (26.50–34.68)	185	38.30 (34.07–42.71)
LRR	492	3	0.61 (0.12–1.86)	0	0 (0.00–0.03)	85	17.28 (14.18–20.88)	29	5.89 (4.11–8.36)
NBR	494	0	0 (0.00–0.93)	0	0 (0.00–0.93)	91	18.42 (15.24–22.09)	49	9.92 (7.57–12.89)
URR	485	60	12.37 (9.72–15.62)	1	0.21 (0.00–1.28)	133	27.42 (23.64–31.56)	71	14.64 (11.76–18.08)
Total	1954	198	10.13 (8.87–11.55)	5	0.26 (0.09–0.62)	456	23.34 (21.51–25.26)	334	17.09 (15.49–18.83)

Using the urine filtration technique, 198 school children of the 1954 children (10.1% prevalence; 95% CI: 8.87–11.55) were positive for *S*. *haematobium*, infections (Table 1). Central River Region (CRR) has the highest prevalence of 27.95% (95% CI: 24.13–32.12) followed by Upper River Region (URR) (12.4%(95% CI: 9.72–15.62)). Lower River Region (LRR) had only 3 positive cases (0.6% prevalence (Table 1). Only 5 cases of *S*. *mansoni* were detected out of the 1954 participants tested using the double Kato-Katz technique. Four of the 5 positive cases were seen in CRR and the fifth was seen in URR (Table 1). This presented a prevalence of 0.3% (95% CI: 0.1–0.6) of intestinal schistosomiasis in the study area. One out of the 5 positive cases had 25 egg/ gram of faeces while the remaining 4 counted only 1 egg/slide.

### Prevalence of intensity of schistosomiasis

No case of *S*. *haematobium* was detected among the 494 participants tested in North Bank Region (NBR). Of the 198 infected children, 2.7% (95% CI 2.07–3.54) had heavy infection and majority of whom were found in CRR (8.3%; 95% CI 6.12–11.10)(Table 2). The mean egg count for *S*. *haematobium* was 17.8 eggs/10 ml urine in CRR, 6.1 eggs/10 ml urine in URR and 0.4 eggs/10 ml urine in LRR (Table 2).

**Table 2:** Infection intensity and mean egg count by urine filtration and Kato-Katz by region.

Region	no. children investigated	no. heavy infected		prevalence of heavy infection % (95% CI)		Mean egg count (95%CI)	
		KK	Filter	KK	Filter	KK	Filter
CRR	483	0	40	0 (0.00–0.95)	8.28 (6.12–11.10)	0.01 (0.00–0.02)	17.83 (11.70–23.95)
LRR	492	0	1	0 (0.00–0.03)	0.20 (0.00–1.26)	0	0.39 (0.00–1.15)
NBR	494	0	0	0 (0.00–0.93)	0 (0.00–0.93)	0	0
URR	485	0	12	0 (0.00–0.95)	2.47 (1.37–4.32)	0.05 (0.00–0.15)	6.07 (2.61–9.53)
Total	1954	0	53	0 (0.00–0.24)	2.71 (2.07–3.54)	0.01 (0.00–0.04)	6.01 (4.24–7.79)

Calculated for all children investigated, irrespective of their infection status

Students were considered to have heavy infection for *S*. *haematobium* if they have at least 50 eggs/10 ml of urine and for *S*. *mansoni* more than 399 eggs/gram of faeces.

### Co-endemicity of *S*. *haematobium* and *S*. *mansoni*

**Fig 2:**
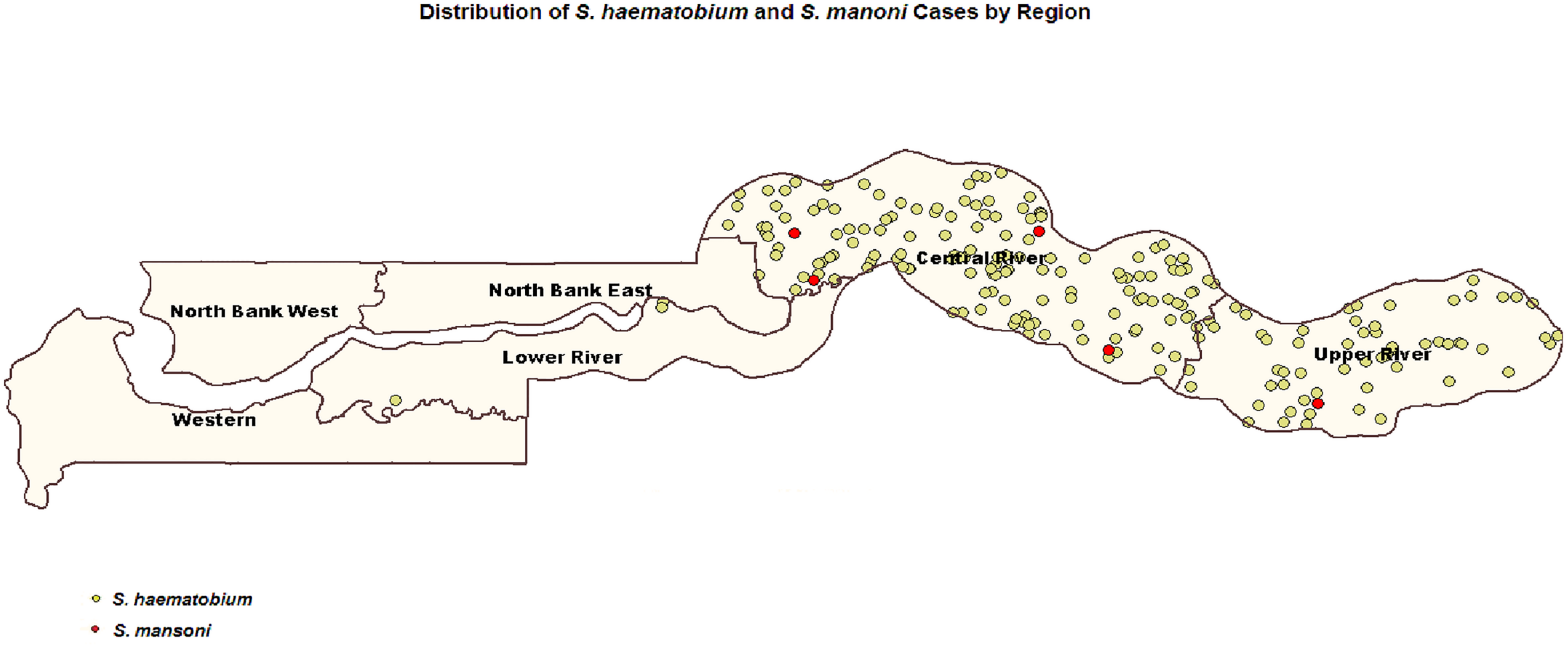
Co-endemicity of *S*. *haematobium* and *S*. *mansoni*.

Three of the 5 participants that tested positive for *S*. *mansoni* were co-infected with *S*. *haematobium*. The existence of these few cases of *S*. *mansoni* in CRR and URR where *S*. *haematobium* is prevalent demonstrated that the two diseases are co-endemic in these regions (Fig 2). The prevalence of antigen to schistosomiasis infection (*S*. *haematobium* and *S*. *mansoni*) tested by POC-CCA was 23.3% (95% CI: 21.5–25.3). The prevalence of micro-hematuria was highest in CRR 38.30% and lowest in LRR with 5.89% (Table 1).

### Sensitivity and specificity of POC-CCA

Using urine filtration as a standard, POC-CCA had a sensitivity of 47.7% in the high endemic regions whilst 66.7% in the low endemic regions(CRR and URR), and 47.98% in all 4 regions. In the high endemic area, 24.2% of children who were egg negative by filtration were CCA positive. In the low endemic area, 17% of egg-negative children were CCA positive (Table 3). The sensitivity of CCA for the schistosomiasis using kato- Katz as standard was 60% (3/5 children). The specificity of the CCA was 75.8%, 82.3% and 79.4% in the high endemic regions, low endemic regions and all 4 regions using filtration as standard, respectively (Table 3). The sensitivity and specificity of POC-CCA using the Dipstick method as the gold standard were 47% and 81.5% in all the participants tested, respectively.

**Table 3:** Sensitivity and specificity of POC-CCA against urine filtration, Kato-Katz and dipstick test techniques by endemicity.

Region	Number tested per region	POC-CCA neg/pos	Filtration—(#/%)	Filtration + (#/%)	Sensitivity/specificity	KK-(#/%)	KK+ (#/%)	Sensitivity/specificity	Dipstick- (#/%)	Dipstick+ (#/%)	Sensitivity/specificity
High endemic region	968	POC-CCA neg	586 (75.8%)	102(52.3%)	47.69/75.81	686(71.2%)	2(40%)	60.00/71.24	572(80.3%)	116(45.3%)54.68/80.34	54.69/80.34
		POC-CCA pos	187(24.2%)	93(47.7%)		277 (28.8%)	3 (60%)		140 (19.7%)	140 (54.7%)	
**Low endemic region**	968	POC-CCA neg	809 (82.3%)	1 (33.3%)	66.67/82.30	810 (82.2%)	0		749 (82.5%)	61 (78.2%)	21.79/82.49
		POC-CCA pos	174 (17.7%)	2 (66.7%)		176 (17.8%)	0		159 (17.5%)	17 (21.8%)	
**All 4 regions**	**1954**	POC-CCA neg	1395 (79.4%)	103 (52.0%)	47.98/79.44	1496 (76.8%)	2 (40.0%)	60.00/76.76	1321 (81.5%)	177 (53.0%)	47.01/81.54
		POC-CCA pos	361 (20.6%)	95 (48.0%)		453 (23.2%)	3 (60.0%)		299 (18.5%)	157 (47.0%)	

## Discussion

There have been increased efforts to develop more rapid and accurate techniques to achieve the WHO 2020 goals and WHA Resolution 65.21. New rapid mapping tools that can be used at the point of care are required to accelerate mapping process in endemic regions towards the achievement of schistosomiasis elimination targets. These tools need to be accurate and sensitive, since prevalence and intensity of infection will decrease as we move towards elimination of the disease. In this study we evaluated the field performance of POC-CCA for prevalence mapping of schistosomiasis at point-of–care for the first time in a country which is endemic for *S*. *haematobium*. A total of 40 schools were mapped across four regions in The Gambia. From the study findings, POC-CCA prevalence was 23.3% (95% CI, 21.5–25.3) which was two times higher than the prevalence based on egg-detection for *S*. *haematobium* 10.1% (95% CI 8.9–11.6) and hundred times *S*. *mansoni* (; 0.26%, (95% CI, 0.09–0.62) respectively.

Sensitivity of POC-CCA against urine filtration technique in detecting *S*. *haematobium in* the four regions was 47.98% which was comparable to the sensitivity in the high endemic regions (47.69%). The specificity of POC-CCA technique was 79.44% for the four regions, 82.30% for the low endemic regions and 75.81% for the high endemic regions. The sensitivity of POC-CCA is generally low in this study, which is in line with findings of previous studies that found POC-CCA not very efficient in detecting *S*. *haematobium* [22]

Using double Kato-Katz as a reference standard for *S*. *mansoni* detection, the sensitivity of the POC-CCA proved to be relatively higher (60.0%) compare to the POC-CCA against urine filtration technique, although few infections were found in the southern region of the country. Coulibaly and others reported a sensitivity of 75.0% for double CCA when compared to four Kato-Katz as reference standard in detecting S. *mansoni* [9]. Also the 2 Kato-Katz positive cases that were reported negative by the POC-CCA both had 1 egg/slide. This low egg count might be the reason they were missed by the rapid test. This is in concordance with previous findings by Coulibaly and others [9], who found the prevalence of *S*. *mansoni* estimated by a single POC-CCA to be 51.7% compared to 20.3% prevalence given by the egg-detection methods. Another study in Ethiopia reported a prevalence of 65.9% by POC-CCA and prevalence of 43.1% by double Kato-Katz in an area of moderate prevalence for *S*. *mansoni* which was similar finding s to another study in Kenya [23,24]. Colley et al also observed higher prevalence based in single POC-CCA than in a single Kato-katz in a study conducted in five African countries in 2013 [25]. Studies employing serological methods have yielded higher prevalence estimates than traditional parasitological methods for diagnosis of schistosomiasis [4, 9, 14, 26]. Furthermore, a meta-analysis found that many studies conclude antigen testing for *S*. *haematobium* yielded poor sensitivity and specificity, however antigen testing was found as an effective tool rapid diagnosis of *S*. *mansoni* [15,23].

According urine filtration and dipstick techniques are more sensitive and specific than the POC-CCA in detecting *S*. *haematobium* infection in high endemic regions. Previous findings had indicated POC-CCA to be less sensitive in detecting *S*. *haematobium* compared to other techniques [26,22].POC-CCA is a rapid, simple and easy-to-use technique and efficient in diagnosing *S*. *mansoni* infection [27]. However, it proved not to be a very useful tool in detecting *S*. *haematobium* in this study as concluded in the meta-analysis of CCA for diagnosis of schistosomiasis [14, 15]. This makes it a less favorable tool for mapping urinary schistosomiasis in endemic countries.

## Conclusion

This study evaluated the potential of the rapid test kit in detecting schistosomal antigens in Lower Basic School going children. A total of 40 schools were surveyed for the prevalence and intensity of urinary and intestinal schistosomiasis. According to the results obtained by the antigen detecting POC-CCA, prevalence of schistosomiasis was two times higher than the prevalence reported by egg-detection methods. Of the four regions in the study, CRR has the highest prevalence of urinary schistosomiasis. From the 1954 individuals sampled, 198 were infected with *S*. *haematobium*. Although The Gambia is known to be endemic for only *S*. *haematobium*, yet 5 subjects were found to harbor *S*. *mansoni*. These 5 individuals were from CRR and URR, which are the high endemic schistosomiasis regions. Three individuals were co-infected with *S*. *haematobium* and *S*. *mansoni*. Sensitivity of POC-CCA kit in detecting *S*. *haematobium* was lower whilst the sensitivity in detecting *S*. *mansoni* was relatively higher. Specificity of the kit was generally higher. The sensitivity of the tool was also observed to be higher in low urinary schistosomiasis endemic regions than in high endemic regions. POC-CCA could be a useful tool for the rapid mapping of intestinal schistosomiasis. However, from the outcome of the current study it may not be an ideal technique for mapping in urinary schistosomiasis endemic regions.

Also, in future evaluations of POC-CCA, other rapid diagnostic test kits can be used alongside in similar large sample size studies. More accurate techniques such as ELISA and PCR should be employed to further confirm the field performance of the POC-CCA.

KK: Kato-Katz
